# The interplay of context factors in hypnotic and sedative prescription in primary and secondary care—a qualitative study

**DOI:** 10.1007/s00228-018-2555-9

**Published:** 2018-09-13

**Authors:** Vivien Weiß, Roland Nau, Gerd Glaeske, Eva Hummers, Wolfgang Himmel

**Affiliations:** 1 Department of General Practice, University Medical Center, Göttingen, Humboldtallee 38, 37073 Göttingen, Germany; 2 Department of Geriatrics, Evangelisches Krankenhaus Göttingen-Weende, An der Lutter 24, 37075 Göttingen, Germany; 3 SOCIUM Research Center on Inequality and Social Policy, University of Bremen, Mary-Somerville-Straße 3, 28359 Bremen, Germany

**Keywords:** Drug utilization, Hypnotics and sedatives, Hospital medical staff, Family physicians, Attitudes of health personnel, Qualitative research

## Abstract

**Purpose:**

Non-medical or contextual factors strongly influence physicians’ prescribing behavior and may explain why drugs, such as benzodiazepines and Z-drugs, are still frequently prescribed in spite of well-known adverse effects. This study aimed to explore which contextual factors influence the prescription of hypnotics and sedatives and to compare their role in primary and secondary care.

**Methods:**

Understanding medical practices as games with specific rules and strategies and performed in a largely habitual, not fully conscious manner, we asked a maximum variation sample of 12 hospital doctors and 12 general practitioners (GPs) about their use of hypnotics and sedatives. The interviews were analyzed by qualitative content analysis.

**Results:**

Hospital doctors’ and GPs’ use of hypnotics and sedatives was influenced by a variety of contextual factors, such as the demand of different patient groups, aims of management, time resources, or the role of nurses and peers. Negotiating patient demands, complying with administrative regulations, and finding acceptable solutions for patients were the main challenges, which characterized the game of drug use in primary care. Maintaining the workflow in the hospital and finding a way to satisfy both nurses and patients were the main challenges in secondary care.

**Conclusions:**

Even if doctors try to act rationally, they cannot escape the interplay of contextual factors such as handling patient needs, complying with administrative regulations, and managing time resources. Doctors should balance these factors as if they were challenges in a complex game and reflect upon their own practices.

**Electronic supplementary material:**

The online version of this article (10.1007/s00228-018-2555-9) contains supplementary material, which is available to authorized users.

## Introduction

Hypnotics and sedatives, especially benzodiazepines and Z-drugs, have well-known adverse effects, such as withdrawal symptoms upon discontinuation, cognitive difficulties, impairment in activities and daily living, and increased risk of falls [1]. They are still frequently prescribed in both primary and secondary care, often as a “quick fix solution” for “nervousness,” “sleep problems,” and other life problems, especially in old age [2–4]. A recent European study [5] found that more than 36% of elderly patients received at least one benzodiazepine during hospitalization. Hypnotics and sedatives are also often prescribed in primary care [6–8].

We can, therefore, assume that hypnotics and sedatives are not always prescribed according to guidelines [9] or the criteria for rational prescribing [10, 11]. Other non-medical or contextual factors seem to influence physicians’ prescribing behavior strongly. Studies have examined these factors, particularly in primary care, where a systematic review and meta-synthesis identified the time and pressure constraints as well as a lack of alternatives, perceptions of patient expectations, and the wish to maintain good doctor-patient relationships as reasons for prescribing these drugs [12]. General practitioners (GPs) seem to consider benzodiazepines the lesser evil due to time constraints in primary care and a perceived lack of alternatives, and they tend to trivialize the side-effects of these drugs [13].

In contrast, we know little about the perspective of hospital doctors or the role of contextual factors in hospitals. A single Australian hospital study performed 25 years ago demonstrated that patient expectations in the case of sleep difficulties result in high rates of benzodiazepine use [14]. However, since physicians’ perceptions seem to be similar in the case of antibiotic prescriptions, including the experience of strong patient expectations [15], we can build on some interesting results regarding the role of contextual factors in hospital physicians’ use of antibiotics. For example, an Australian antibiotic study following Bourdieu’s theory of practice [16] described antibiotics used as a “game” within the habitus of the social world of the hospital [17]. Thus, medical practices can be understood as games, driven by specific rules, which are taken for granted, rather than being driven by, for example, what is recommended in the therapeutic guidelines. For example, the acute hospital context of the Australian study [17] resulted in a game that was geared towards concordance with peer practices, managing time pressure, and protecting patients by reducing immediate risks, among other factors. In contrast, the threat of antimicrobial resistance was of limited significance for hospital doctors; as a result, their prescription of antibiotics was sub-optimal as a logical consequence of the contextual factors of the hospital.

The key question of this study is which contextual factors influence the prescription of hypnotic and sedative drugs, with a special focus on benzodiazepines and Z-drugs. Since the role of contextual factors may be better understood if we compare distinct spheres within health systems with different rules, norms, and expectations, we studied hospital doctors’ and GPs’ use of hypnotic and sedative drugs.

## Methods

### The project

This study is part of a larger mixed-methods project addressing the use of hypnotics and sedatives at the interface of hospital care and general practice [18]. Ethical approval was obtained from the Ethics Committee of the University Medical Center Göttingen (ref. number 25/2/15).

### Research concept and design

Understanding medical practices as games with specific rules and strategies and performed in a largely habitual, not fully conscious manner [16], we used qualitative methods to explore doctors’ prescribing habits and to generate broad descriptions of the prescribing processes, motives, and experiences in primary and secondary care. Semi-structured interviews and a qualitative content analysis based on the method of deductive and inductive coding should help to understand better the influence and interplay of the contextual factor on the ‘game’ of prescribing hypnotic and sedative drugs.

### Participant selection

The selection of participants was based on the principle of maximum variation’ sampling [19] and occurred in two steps: (1) We invited all hospital doctors in the participating regional general hospital via email and within the internal news service. After an additional invitation of a subsample of 21 hospital doctors according to the hierarchical position, gender, and department, a total of 10 doctors agreed to participate. We also invited per letter, fax, and telephone contact a sample of 46 GPs stratified by age, gender, and urban vs. rural setting from Lower Saxony and Northern Hesse, Germany; 10 of them agreed to participate. (2) After a preliminary analysis of the first interviews in both settings, we deliberately addressed some more doctors with characteristics that seemed to be important to obtain and understand the full scope of experiences.

Thus, two more female hospital doctors from internal medicine and general surgery of the participating hospital and two younger GPs were invited. Recruitment continued until data saturation was reached, i.e., no further information emerged.

### Data collection

VW conducted all of the interviews between January 2015 and October 2016. They lasted half an hour on average (range = 17 to 56 min) and were audio-recorded and transcribed verbatim. VW had no relationship with any of the participants. The participants were informed about the project and gave written consent. Except for one telephone interview, we conducted all interviews face to face in the hospital or the participants’ practices.

The interviews started with an open question to prime physician’s memory and encouraged them to report own experiences (and not only professional attitudes) (see Table 1 and Appendices 1 and 2). An interview guide provided a flexible framework for exploring those experiences with drug prescriptions that were not spontaneously reported in the participants’ initial narratives [20].

**Table 1:** Opening question and topics of the questioning phase of the interview guidelines

Opening question*	Topics of the questioning phase
“As you already know, our study covers the prescription of sedatives and hypnotics for elderly patients. Scientific and lay journals have published articles that are critical of this subject in recent years. However, very little is known about this subject, particularly from the perspective of the hospital doctors/general practitioners. Please try to remember the last few cases in which you prescribed sedatives or hypnotics and tell me what prompted you to do this.”	Pharmaceutical guidelines
	Alternative treatment options
	Doctor-patient relationship
	Experience of particular or critical situations
	Need for improvement
	Interface hospital/general practice

*Same opening question for GPs and hospital doctors

The interview guide was developed and tested in four pilot interviews with GPs and hospital doctors who were not included in the sampling frame. The topics of the guide (see Table 1 and Appendices 1 and 2) were drawn from current literature and a previous quantitative survey [21] and were informed by the experience of clinical members of the research team.

### Analysis

We analyzed all the interviews using the principles of qualitative content analysis, incorporating data- and concept-driven approaches [22] (see the flowchart of the data analysis in Appendix 3). Coding was mainly conducted by VW, WH, and a research assistant, supported by two workshops with researchers from other disciplines and regular meetings of an inter-professional research team with expertise in hospital geriatrics, family medicine, nursing science, health services research, and medical sociology, to validate ratings and achieve consensus. Transcripts were read and relevant passages identified, the paraphrased analysis units were reduced through bundling and sorted into categories. The categories were ordered following the definition of “external context factors,” as defined by Helman [23], including the physical place or setting, equipment, atmosphere, type of relationship, status, and type of information. In a final step, the categories were summarized at a higher level as “context factors,” using Bourdieu’s concept of “theory of practice” [16]. Thus, the interplay of contextual factors in the medical practice could be described and their role in primary and secondary care compared. All main categories (defined as context factors) were illustrated using representative quotations. Also, all subcategories are illustrated by exemplary quotes (see Appendix 4). MAXQDA 12 was used for computer-assisted qualitative data analysis [24] (see Appendix 5).

Intercoder reliability analysis was not determined. The validity and usefulness of themes and subthemes were regularly evaluated by the research team to ensure consistency and coherency [25]. All quotations in this article were translated into English by a professional translation service. This methods and results of the study are reported according to the COREQ guidelines (for additional information see Appendix 6) [26].

#### Data availability

The Ethics Committee of the University Medical Center Göttingen does not allow us to share the dataset with other parties. For questions about the dataset, please contact the corresponding author.

## Results

### The sample

The sample consisted of 12 hospital doctors (3 females) with an average age of 37 years and 12 GPs (5 females) with an average age of 56 years. The hospital doctors worked in different departments: geriatrics (three), emergency medicine (two); internal medicine (three); general surgery (two), and trauma surgery (two); one of the participants was a chief physician, six were senior physicians and five assistant physicians. Seven of the GPs worked in urban areas (two females) and five in rural areas (three females).

### Emerging categories

The hospital doctors and GPs frankly described many different situations and problems in which they used hypnotics and sedatives or felt that their use would be expected. We identified eight major context factors such as “patient groups” or “aims of management”. They are described in the following and illustrated by representative quotes. Additional quotes, together with their subcategories, can be seen in Appendix 4.

## Patient groups

### Primary care

The GPs described different groups of primary care patients who needed or asked for hypnotics or sedatives: patients in acute life crises (such as the death of a relative, divorce, exam situations or job loss); geriatric patients with insomnia or psychiatric problems; and rather frequently, postmenopausal women (see quote). In particular, GPs felt that patients experiencing acute life crises and associated sleeping problems asked for hypnotics or sedatives if they perceived that the usual methods (e.g., prescription-free medications) were insufficient. Another large group that requested hypnotics or sedatives comprised long-term dependent patients.

Women, particularly postmenopausal women, prone to sleep disruption are sensitive to sleep quality. To not be able to sleep is a surprising experience for many women as estrogen production declines. Palpitations, sleep disorders, etc. are frightening because they are often still working or parenting. (GP6, urban, female: P23)

### Secondary care

The hospital doctors reported that almost all patients asked for hypnotics or sedatives to obtain a restful sleep during a hospital stay.

It's the classic complaint; almost all patients moan that they can’t sleep properly here [in the hospital], it’s a daily occurrence that a patient simply asks can I have something to help me sleep at night. (HD6, internal medicine, assistant physician, male: P13)

## Patient demand

### Primary care

The GPs felt pressured by patients who expected hypnotics or sedatives because of unspecific worries about sleep problems (see quote); unrealistic expectations regarding sleep (for example, expecting to sleep 8 to 10 h daily); serious diseases; acute life crisis; or the expected refill of previous prescriptions. Particularly in the first two instances, the physicians perceived patients as deeply insulted and disappointed if their expectations were not met.

In some cases, it’s like talking to a wall: (the patients say) I need my prescription and if I don’t get it then I’ll jump out of the window (...) in such circumstances some quite drastic and desperate [patient], statements can be heard. (GP12, urban, male: 24)

### Secondary care

The hospital doctors frequently described patients who expected a relaxed hospital stay and demanded hypnotics or sedatives because of unfamiliar surroundings and strange noises (see quote). Moreover, seriously ill patients often suffered from sleep problems because of therapeutic interventions and consequently expected hypnotics or sedatives. In some cases, patients expected the continuation of long-term medication use.

Many patients simply want to be given something because they can’t sleep well here [in the hospital]; there’s the oxygen pump running and the nurse constantly coming in. They feel their nightly rest is very disrupted and say, as I’m here [in hospital] can I at least have my peace and quiet. (HD6, internal medicine, assistant physician, male: P15)

## Aims of management

### Primary care

The GPs followed two aims when prescribing hypnotics and sedatives. First, they tried to retain their patients. They considered it essential to have a good relationship with their primary care patients and to fulfill their expectations, even if it required prescribing hypnotics and sedatives that were not pharmacologically necessary. Sometimes, they apparently used this strategy to retain their patients, especially those who were privately insured. Controlling the use of hypnotics and sedatives was the second aim—and challenge—for the GPs. Some interviewees tried to find individual solutions (e.g., interval treatment) to avoid dependency, help primary care patients benefit from the efficacy of the drugs and meet patients’ desires for effective treatment.

Of course, I always try to make it clear, that frequent use of sleeping pills may diminish their ability to work over time. And I try to convince patients to adopt an interval treatment simply to retain the effectiveness of the medication. (GP12, urban, male: 24)

### Secondary care

For many of the hospital doctors, the use of hypnotics and sedatives for sleeping problems was not a matter of significant concern. For example, surgeons and internists considered the reasons for admission, not the use of sleeping pills, as a mandate to act (see quote). Hospital geriatricians considered a restful sleep important for recovery and the realization of rehabilitation goals.

Well, in the surgical ward sleeping pills are not significant. It’s more of a fringe area for us. (HD10, general surgery, senior physician, male: P8)

## Time resources

### Primary care

The GPs complained about time pressure when treating patients who repeatedly asked for hypnotics or sedatives (see quote). They felt overburdened by the need to contain dosages, consider non-medical problems, and recommend alternative treatments. The majority mentioned that the costs of these lengthy consultations were not covered by statutory health insurance unless the physician had supplementary training in psychosomatic medicine and could label the consultation as psychosomatic treatment, a particular case in Germany.

So, in the scenario, a patient comes and says I can’t sleep, and I simply prescribe a sleeping pill, that’s not what I do, I never have (...) But it takes a lot of time that the system does not have. (GP7, rural, female: P92)

### Secondary care

Almost all the hospital doctors said that the handling of hypnotics or sedatives depends on the work shift (“regular” versus “on call”). During the regular day shift, ward physicians appreciated the opportunity to act in a patient-oriented way. Physicians on call felt pressured, did not know all of the patients and tried to solve issues quickly—even if the solution was not always according to their principles.

(...) Sometimes I do it without questioning because of the pressure, the stress and the need to somehow get through the day. Also things are done against better judgment. (HD3, intensive unit, assistant physician, male: P39)

## Prescribing options

### Primary care

The GPs mentioned two prescribing options for primary care patients with statutory insurance coverage: prescribing these drugs at the expense of the statutory health insurance (SHI) or, especially in the case of long-term prescriptions, prescribing them outside SHI coverage so that patients must pay for the drugs. (Note: normally, drugs are covered by SHIs which pay pharmacies for reimbursed drugs with some co-payment by patients (max. 10 €). However, any physician can also provide private prescriptions even for SHI-insured persons. Because these prescription forms are not submitted for reimbursement, the prescriptions have to be fully paid for by the patient. More importantly, information is not recorded in SHIs claims data. Therefore, private prescriptions are a “black-box” for research and quality assurance [27]). The majority reported that they used the latter option because they feared recourse claims by the SHI and hoped to cap their prescribing budget. Another reason for the latter strategy was to encourage personal responsibility and self-care among patients and to demonstrate that there is no medical need for the prescription.

Interviewer: Are there any other reasons why you prescribe these drugs outside the GKV Act?

GP13 (urban, male: P28): Yes, on the one hand, it is a legal requirement, and on the other, the patient should be reminded, quite clearly, that this is not medically necessary*.*

### Secondary care

The hospital physicians, junior doctors, in particular, reported that hypnotics and sedatives were mostly prescribed as prn medications [“pro re nata”: meaning “as needed” or “as the situation arises”]. Only if they expected that a persistent problem could become a long-lasting problem would they prescribe these drugs as regular medications.

If it is likely that it is really a long-term problem, going on over some time [as a chronic prescription], but if I do not know the patient it is primarily based on need. (HD6, internal medicine, assistant physician, male: P29)

## Setting-dependent risk assessments

### Primary care

The main problem for the GPs was dealing adequately yet efficiently with requests for repeat prescriptions. For example, they described how primary care patients asked the practice nurses for a repeat prescription for a benzodiazepine or a similar drug. The practice nurses often prepared a prescription form and left it to the doctor to identify inappropriate prescriptions. In the chaos of a busy practice, prescriptions may be signed uncritically, and primary care patients may receive the prescription without a consultation. Some GPs considered the risk of falling more dangerous than the risk of drug dependency because they cannot ensure care for their patients 24 h a day.

(Physician) I have the impression that we try not to prescribe these medications because they lead to falls. That means that addiction is not really the reason. (GP10, urban, female: P35)

### Secondary care

The hospital doctors considered that prescribing hypnotics and sedatives as prn medications may prompt nurses to dispense them to cope with stressful situations during night shifts, without seeking medical advice or considering pharmaceutical needs. Furthermore, some of the hospital doctors had encountered situations in which prn-dispensed hypnotics or sedatives caused in-house emergencies (see quote). Paradoxical reactions were also mentioned as a risk. Surgeons, in particular, noted the risk of falls in the hospital. Drug-induced falls were perceived as not rare and as a potential cause of infections or periprosthetic fractures. Furthermore, the hospital doctors were concerned that they might overlook people at risk of experiencing withdrawal symptoms during the hospital stay.

[In the notes] a prescription medication is written … but no maximum dose is noted but that the patient cannot sleep, and another dose is slipped in, (...) sometimes even without being documented, (...) it’s also the case that nurses take it upon themselves to issue these substances. (HD3 intensive unit, assistant physician, male: P105)

## The role of nurses

### Primary care

Practice nurses are a key link in the prescription chain. Not only are they responsible for communicating patients’ wishes for a prescription to the GPs but they also prepare repeat prescriptions (of any kind) for signature and may thus unintentionally enable primary care patients to receive hypnotics or sedatives without proper consultation with the GP.

I think the greatest danger is always that they’ve been given benzodiazepines twice, and somehow this is then included with the great number of prescriptions passed to them over the counter in the reception area without us General Practitioners having talked with the patients. (GP7, rural, female: P144)

### Secondary care

In cases of sleep problems, nurses are the main contact persons for inpatients. The hospital doctors reported that only in a few instances patients directly asked for hypnotics or sedatives (e.g., during medical rounds). In contrast, doctors reported that nurses manage and control the access to these drugs. At times, nurses even dispense prn-dispensed hypnotics and sedatives on their initiative; although they are not authorized to do so (see quote). Alternatively, hospital doctors sometimes prescribed hypnotics and sedatives for agitated patients to reduce the workload of the nursing staff.

Legally, the doctor on duty must have seen the patient to judge whether they are so restless and delirious, that they need a prescription medication (...). In reality, it is unfortunately sometimes the case that a prescription medication has been specified and all is written down from the previous day or nights, and that it is then administered by the nurses without further consultation of the doctor. (HD7, internal medicine, senior physician, male: P153)

## The role of colleagues

### Primary care

The GPs indicated that they sometimes had to handle prescriptions from their colleagues or predecessors. In these cases, they felt left alone to convince the primary care patient of the pros of alternative treatments or more adequate medications. If patients insisted on maintaining their prior drug regimen, the GPs often continued the prior prescription without further discussion (see quote).

Of course, there are some cases where we simply continue to prescribe, where we weren’t the ones that made the first prescription (...) there are many extensions to prescriptions from previous doctors. (GP3, urban, male: P19)

### Secondary care

One physician reported that colleagues on duty sometimes prescribed hypnotics or sedatives other than the one he prefers, obviously without any further consequences.

(...) well, it does happen that I think, ah, what was he thinking here, there’s no need for a prescription of Benzo, or something similar, but this is fairly infrequent. (...). (HD3, intensive unit, assistant physician, male: P69)

Interestingly, the GPs and hospital doctors appeared to act independently of one another. They usually did not feel a necessity to share information on their patients’ use of hypnotics or sedatives, including indications, dosage, former adverse effects, or the risk of dependency. Some of the GPs considered this issue—compared, for example, to cardiovascular diseases or diabetes—too trivial. Some hospital doctors appreciated an immediate information exchange (e.g., through personal contact via telephone) with their primary care colleagues, but not in the case of a short intake of hypnosedatives during hospitalization. Furthermore, they indicated that primary care prescriptions for hypnotics or sedatives were usually continued at the hospital because they thought a hospital stay was not the best time for withdrawal.

## Discussion

The hospital doctors’ and GPs’ handling of hypnotics and sedatives and their experiences differed markedly. Their game of drug use, as Broom and colleagues put it [17], was influenced by a variety of context factors and their interplay in the fields of primary and secondary care, such as different patient groups and their demands, aims of management, time resources, setting-dependent risk assessments, and the role of nurses and peers in primary and secondary care.

### Comparison with literature

Several studies have investigated patient demand as a contextual factor for drug prescriptions [28]. GPs, in particular, perceive a patient’s demand as the main reason for prescribing antibiotics [29, 30]. The results of our study confirm that primary care patients’ demand has an important role in the prescribing of hypnotic and sedative drugs. Patient demand was also a substantial factor in hospitals, but ironically, since so many patients expected “something to sleep”, The hospital doctors seemed to perceive this demand as usual care and not as a conflict, in contrast with their primary care colleagues.

GPs are strongly influenced by regulations (“drug budget”) [31] that require the cost-efficient prescription of drugs and limit prescriptions for hypnotics and sedatives at the expense of the statutory health insurance to 4 weeks (if no reason for more prolonged  use is given). These regulations are irrelevant for hospital doctors.

Setting-dependent risk assessment was a further context factor. Initially and intuitively, we thought that the risk of falls might be of less significance for GPs than for hospital doctors. In contrast to this assumption and the reports of other studies [32, 33], our results demonstrate that the GPs were more aware than the hospital doctors of the risk of falling [34, 35]. This risk was sometimes perceived as even higher than the risk of tolerance or dependence. In contrast, the hospital doctors also emphasized the challenge of avoiding withdrawal symptoms or paradoxical reactions.

Time resources also influenced the handling of hypnotics and sedatives: the hospital doctors, especially assistant physicians, expressed their discontent with acting under time pressure, sometimes against their professional principles. This is in line with a German study of the associations between job demands and perceived quality of care [36]. Time constraints in connection with lack of alternatives have also been identified as an important factor in GPs’ experiences and perceptions with prescribing benzodiazepines [13, 37]. Similarly, resource limitations and the lack of alternatives—or the lack of awareness of alternative options—were reasons for psychiatrists in New Zealand [38] to continue hypnosedatives and to consider guidelines for the use of these drugs unhelpful.

Nurses and medical peers also influenced the prescription process. While “nurses” are known to be important to the prescription of antibiotics [39, 40], we found that they helped to meet patients’ demands on hypnosedatives without consulting a doctor. According to a secondary data analysis of the French health insurance database, more than one third of Z-drug prescriptions were issued without a face-to-face consultation [41]. For hospitals, recent studies have described the distribution of as-needed medications, especially by mental health nurses [42] and for hospitalized psychiatric patients [43]. Older studies [44, 45] emphasize nurses’ influence in general hospitals as well. Some assistant physicians in our study reported a liberal use of hypnotics and sedatives by nurses. This is in line with results from other studies, e.g., nurses’ “automatic” use of benzodiazepines in nursing homes [46]. One reason may be that nurses do not consider these medications to be a risk that outweighs the perceived benefits [46, 47]. More importantly, instead of blaming nurses for this behavior, we would like to emphasize that nurses—even if they are pharmacologically best trained—will still be caught between the demands for fulfilling high standards of patient care and for keeping the hospital running, especially during the night shift [45]. Any measures to change this situation should help nurses (and doctors) by acknowledging and easing this catch-22 situation.

Our GPs felt pressured to prescribe hypnotics and sedatives when primary care patients had received these drugs from other GPs or a former practice owner [48]. Hospital doctors, too, felt influenced by other colleagues, but not to the extent described in other studies [49, 50]. According to our results, ward physicians and physicians on call sometimes disagreed regarding the use of drugs without discussing these discrepancies with each other. The authors of a study about antimicrobial prescription described such behavior as “prescribing etiquette” to illustrate the influence of “cultural rules” on prescribing decisions [49].

Improving the quality of care at the medical primary-secondary care interface is considered a wider concern in most countries. Sampson et al. (2016), for example, found in their qualitative interview study with Scottish GPs and hospital specialists, a will and determination to improve things for both themselves and the benefit of patient care [51]. In our study, however, in the case of hypnosedatives, we were confronted with an openly articulated mutual disinterest to give this issue a high priority, probably because it was not the reason for admission.

### Meaning of the study

- The concept of game [17], shortly introduced in the “Introduction” section, may help to understand better how the variety of contextual factors and their interplay determine, among other issues, the prescribing of hypnotics and sedatives in primary and secondary care and why the game is differently played in general practices and hospitals.
- The game in primary care seems to be geared towards a tradeoff between complying with guidelines or regulations and finding ways to address primary care patient demands within a reasonable time frame. Thus, GPs use strategies such as out-of-pocket prescriptions or interval treatment with drug holidays as ways to meet patient needs or express empathy and to hide the feeling that, to put it diplomatically, they are powerless to intervene effectively. Peers and practice nurses have a significant role in this game, especially in promoting repeat prescriptions that are sometimes not medically indicated.

The game in secondary care was geared towards keeping things in the hospital on track and finding a way to satisfy both nurses and inpatients. In the interviews, hospital doctors weighed—often in an exemplary manner—the potential benefits of hypnotics and sedatives against potential adverse effects. At the same time, they reported the liberal use of these drugs in the hospital and mentioned the role of work shifts, time resources, and nurses’ demands, typically resulting in prn prescriptions with nurses becoming responsible for their administration.

In the case of hypnotics and sedatives, the games in the field of primary and secondary care are played with almost no communication between the two groups of doctors, apart from usually listing the drugs administered in the hospital in the discharge letter. Since approximately 1% of patients discharged from acute care hospitals became chronic benzodiazepine users [52], we were surprised that hospital doctors and GPs do not see more need to cooperate and exchange information. Different requirements and characteristics of contextual factors in both fields of practice and a lack of understanding for each other’s work environments make cooperation difficult—as shown in a recent British study [53]. One solution, to support the exchange of medicine reconciliation across the primary and secondary care sectors in Germany is to motivate patients to allow all healthcare providers to store medication-related information via the electronic health card. However, this requires a good doctor-patient relationship [54].

The notion of drug prescribing as a game may provoke the impression that doctors do not prescribe rationally [11]. This is not the intention of the study. Rather, it should become clear that even if doctors try to act rationally, they cannot escape the interplay of contextual factors. This allows us to move beyond exhortation and accusation as means of producing a behavioral change, as MacDonald et al. [38] put it, but to understand prescribing behavior foremost as a matter of contextual factors in the respective medical settings [55].

### Strengths and weaknesses of the study

Comparing the views and experiences of hospital doctors and GPs allowed us to determine the importance of contextual factors and their interplay for the prescription of hypnotics and sedatives more specifically than former studies focusing on one setting only. An open-interview approach turned out to be suitable to receive deeper insights and to detect unexpected practices that would otherwise not be reported. Furthermore, the application of this approach made it possible to understand practices in the context of different framework conditions from the actors’ subjective perspective [20]. However, while maximum variation sampling and in-depth interviews ensure the transferability of the results to similar constellations, the generalizability of the results is limited, due to the number of participants.

The data analysis considered only external contextual factors [22]. Data regarding the doctors’ background, e.g., their social, cultural, religious backgrounds or medical training and knowledge, was not systematically collected. These factors should be considered in future studies.

Physicians from other disciplines, e.g., sleep or emergency medicine, may have a different view regarding the use of sedatives and hypnotics and other aspects of the game, especially if they have to handle acute and critical situations. For example, the short-term use of benzodiazepines in acute sleep disorders with severe impairments in everyday life, preoperatively, for cramps or fear or panic attacks is considered useful [56]. However, even in these rare cases, everything should be done to prevent short-term use from being prolonged into long-term use. These facts are not necessarily contradictory to our assessment since we were interested in the everyday handling of sleep problems during hospitalization, not critical and severe situations in primary and secondary care.

## Conclusion

Comparing primary and secondary care, we could show how strongly context factors influence doctors’ use of hypnotic and sedative drugs. Context factors—such as patient demands, time frame, the role of nurses and colleagues—often appear to be more important than pharmacological criteria or medical guidelines; they create, at least sometimes, a barrier against guidelines adherence.

The use of the game concept prevents us from the illusion that the problems detected in this study can be easily solved through CME in pharmacology, stewardship initiatives, or treatment guidelines. We obviously face an issue of action rather than of knowledge, or—in other words—the realization of explicit knowledge is influenced by tacit, practice-based knowledge, rest on contextual factors. Interventions to implement evidence-based knowledge should meet the particular situation of physicians and nurses, make the game transparent, make aware of the role of different context factors, remind the “players” of their code of ethics and encourage physicians and nurses to reflect on their practices [52]. In situations where context matters, the Framework for Complex Interventions or the PARIHS Framework recommend to invite all relevant stakeholders, in our case especially GPs, hospital physicians, nurses, and the practice staff as well as the managerial and administrators and discuss how the prescription and use of hypnotics and sedatives can be reduced. Any interventions, developed in such a participatory approach should address both the actions and strategies of the individuals involved as well as the organizational structures of their professional working environment, including the context, in which individuals and teams are bounded by professional issues and communication challenges [18, 57, 58].

One big challenge during a hospital stay is to avoid the initiation and short-term use of hypnotics and sedatives and to prevent drifting into long-term use [59]. Vice versa, the hospital stay could be an opportunity to address problems of drug use that otherwise remain undetected. Even if problems with hypnosedatives are not the main reason for admission, all professionals involved should handle the use of these drugs as a common focus due to patient safety.

The study supports the need to examine multiple factors within the knowledge to action process. One promising and to date neglected approach could be better and active integration of hospital nurses and practice staff as important context factors for the use of hypnotics and sedatives.

## Electronic supplementary material

ESM 1 (DOCX 21 kb)

ESM 2 (DOCX 21 kb)

ESM 3 (DOCX 43 kb)

ESM 4 (DOCX 33 kb)

ESM 5 (DOCX 367 kb)

ESM 6 (DOCX 27 kb)
