# Supplementary material for: The interplay of context factors in hypnotic and sedative prescription in primary and secondary care—a qualitative study
Source: Eur J Clin Pharmacol. 2018 Sep 13;75(1):87–97. doi: 10.1007/s00228-018-2555-9 (PMC6326988; doi:10.1007/s00228-018-2555-9)
Supplement: Supplementary file 1 — (DOCX 21 kb) [file 228_2018_2555_MOESM1_ESM.docx]

**Appendix 1: Interview guideline for open interviews with general practitioners**

**Main Narration Code: __________**

**Opening question**

As you already know, our study covers the prescription of sedatives and hypnotics for elderly patients. Scientific and lay journals have published articles that are critical of this subject in recent years. However, very little is known about this, particularly from the perspective of the general practitioners. Please try to remember the last few cases in which you prescribed sedatives or hypnotics and tell me what prompted you to do this.

Please take your time to recall these situations. It would be helpful if you could describe some details. I am going to simply listen and take notes and refer back to them at some later time.

**Questioning Phase**

**Interface Hospital/General Practice**

It probably happens frequently that patients are given sedatives or hypnotics in hospital. Patients may report favourably about these medications and, directly or indirectly, expect you to prescribe the same medications. Please tell me about the last time where this was the case and describe how it went.

**Pharmaceutical Guidelines**

It appears that in recent years patients have increasingly been prescribed sedatives and hypnotics on private prescriptions. Maybe you are familiar with this phenomenon yourself or have heard of it from colleagues. Please tell me about your experience with this.

**Alternative Treatment Options**

Instead of using sedatives and hypnotics, alternative and complementary treatment options are available. Please tell me whether such treatments would be an option for you, and if so, why.

In which situations do you prefer to use these treatment options?

**Doctor-Patient Relationship**

What roles do behavior and personality of a patient play in your decision about whether to prescribe sedatives or hypnotics?

There may have been a situation when you felt you were influenced by the patient. Please tell me about this.

**Experience of Particular or Critical Situations**

There must be difficult or critical situations in connection with prescribing sedatives or hypnotics in your daily routines. Please try to remember them and describe them for me.

**Need for Improvement**

What areas do you see that need improvement in connection with prescribing sedatives or hypnotics?

**Conclusion**

Is there anything you would like to add to our conversation so far in connection with sedatives and hypnotics?

| **Interviewer** |  | |
| --- | --- | --- |
| **Date** |  | |
| **Place of communication** |  | |
| **Gender** | □ male | □ female |
| **Age** |  | |
| **License to practice since** |  | |
| **Employed as** | □ Head of a family practice  □ Assistant doctor in training  □ Employee in family practice | |
| **Place of practice** | □ urban area  □ rural area | |
