# Supplementary material for: The interplay of context factors in hypnotic and sedative prescription in primary and secondary care—a qualitative study
Source: Eur J Clin Pharmacol. 2018 Sep 13;75(1):87–97. doi: 10.1007/s00228-018-2555-9 (PMC6326988; doi:10.1007/s00228-018-2555-9)
Supplement: Supplementary file 2 — (DOCX 21 kb) [file 228_2018_2555_MOESM2_ESM.docx]

**Appendix 2: Interview guideline for open interviews with hospital doctors**

**Main Narration Code: ___________**

**Opening Question**

As you already know, our study covers the prescription of sedatives and hypnotics for elderly patients. Scientific and lay journals have published articles that are critical of this subject in recent years. However, very little is known about this subject, particularly from the perspective of the hospital doctors. Please try to remember the last few cases in which you prescribed sedatives or hypnotics and tell me what prompted you to do this.

Please take your time to recall the situations. It would be helpful if you could describe some details. I am going to simply listen and take notes and refer back to them at some later time.

**Questioning Phase**

**Interface Hospital/General Practice**

When patients have been treated repeatedly with sedatives or hypnotics in a hospital and are then discharged, is the doctor responsible for further treatment made aware of this? How is this done, and is it effective?

Do you as hospital doctor have other points of contact with general practitioners in connection with the prescription of sedatives and hypnotics?

**Alternative Treatment Options**

Instead of using sedatives and hypnotics, alternative and complementary treatment options are available. Please tell us about your experience with these treatment options and whether you recommend for instance benzodiazepines and Z-substances as alternatives.

**Doctor-Patient Relationship**

What roles do behavior and personality of a patient play in your decision about whether to prescribe sedatives or hypnotics?

There may have been a situation when you felt you were influenced by the patient. Please tell me about this.

**Experience of Particular or Critical Situations**

There must be difficult or critical situations in connection with prescribing sedatives or tranquillisers in the daily routines in the hospital. Please try to remember them and describe them for me.

**Need for Improvement**

What areas do you see that need improvement in connection with prescribing sedatives or hypnotics?

**Conclusion**

Is there anything you would like to add to our conversation so far in connection with sedatives and hypnotics?

| **Interviewer** |  | |
| --- | --- | --- |
| **Date** |  | |
| **Place of communication** |  | |
| **Gender** | □ male | □ female |
| **Age** |  | |
| **License to practice since** |  | |
| **For how many years in the current job** |  | |
| **Employed in department** |  | |
| **Number of beds in the department** |  | |
