# Supplementary material for: The interplay of context factors in hypnotic and sedative prescription in primary and secondary care—a qualitative study
Source: Eur J Clin Pharmacol. 2018 Sep 13;75(1):87–97. doi: 10.1007/s00228-018-2555-9 (PMC6326988; doi:10.1007/s00228-018-2555-9)
Supplement: Supplementary file 3 — (DOCX 43 kb) [file 228_2018_2555_MOESM3_ESM.docx]

**Appendix 3: Flow Chart of the data analysis process**

Step 2

Paraphrasing analysis units.

Step 3

Read the transcripts. Writing memos.

Step 4

Identification of relevant analysis units.

Step 1

*If you are interested in the steps 1, 3, 4, 5, 6, 7 the material can be requested via the corresponding author (in German).

**Picture 1**: Flow chart of the data analysis process*

Integration into the concept „Theory of practice“.

Step 8

Review of the summary categories.

Step 7

Order of categories according to the concept of external context factors, as defined by Hellmann.

Step 6

Inductive identification of categories on the main material.

Step 5

Reduction through bundling, construction, integration of paraphrases.
