# Supplementary material for: The interplay of context factors in hypnotic and sedative prescription in primary and secondary care—a qualitative study
Source: Eur J Clin Pharmacol. 2018 Sep 13;75(1):87–97. doi: 10.1007/s00228-018-2555-9 (PMC6326988; doi:10.1007/s00228-018-2555-9)
Supplement: Supplementary file 4 — (DOCX 33 kb) [file 228_2018_2555_MOESM4_ESM.docx]

**Appendix 4: Main categories, subcategories and exemplary quotes**

**Table 1:** Main categories, subcategories and exemplary quotes, identified from the semi-structured interviews with hospital doctors and general physicians.

| **Primary Care** | **Secondary Care** |
| --- | --- |
| **Main category: Patient groups** | |
| - **Patients in acute life crises**   Quote: *Just anything at all that currently throws the patient out of their usual patterns of behavior and where it's necessary. Might also be in connection with trouble at work, for instance*. (GP13 male: P14)   - **Geriatric patients**   Quote: *It's not just young people asking for sedatives, but the elderly, too, who sit on their sofas the whole day long or had their little nap in front of the telly during their favorite evening show, and who are then surprised that they can't sleep 8 hours on the trot during the night.* (GP11 male: P27)   - **Postmenopausal women**   *Quote 1: Women, particularly postmenopausal women, prone to sleep disruption are sensitive to sleep quality. To not be able to sleep is a surprising experience for many women as estrogen production declines. Palpitations, sleep disorders, etc. are frightening because they are often still working or parenting.* (GP6 female: P23)   - **Long-term dependent patients**   Quote: *It's more common here for sleeping pills that some patients are used to taking the pills. And that over a longer time, even years, and these patients just continue being issued their sleeping pills.* (GP 13 male: P2) | - **Almost all inpatients to obtain a restful sleep during a hospital stay**   *Quote 2: It's the classic complaint, almost all patients moan that they can't sleep properly here [in the hospital], it's a daily occurrence that a patient simply asks can I have something to help me sleep at night* (HD6 male: P13) |
| **Main category: Patient demand** | |
| - **Unspecific worries about sleeping problems**   Quote: *It's quite frightening for many people - you have to sleep, otherwise you'll get sick, otherwise, this and that will happen, and that is very frequently the motivation that urges the patients to make an appointment and ask for help.* (GP 6 female: P15)   - **Unrealistic sleep expectations**   Quote: *But sometimes it's even the case that they go to bed at 8 pm and are truly astonished that they're not sleeping through until 6 am, and that just doesn't work, and then sometimes they go to bed a little later*. (GP10 female P:57)   - **Expected refill of previous prescriptions**   *Quote 3: In some cases, it's like talking to a wall: (the patients say) I need my prescription and if I don't get it then I'll jump out of the window (...) in such circumstances some quite drastic and desperate [patient], statements can be heard..* (GP12 male: 24) | - **Expectation of a relaxed hospital stay**   Quote: *And they (the patients) say, well, when I'm here, I just want to have my peace and quiet and really restore my health, see, and I'd like something to help me sleep better.* (HD6 male: P15)   - **Disturbed sleep because of unfamiliar surroundings**   *Quote 4: Many patients simply want to be given something because they can't sleep well here [in hospital]; there's the oxygen pump running and the nurse constantly coming in. They feel their nightly rest is very disrupted and say, as I'm here [in hospital] can I at least have my peace and quiet.* (HD6 male: P15) |
| **Main category: Aims of management** | |
| - **To retain their patients**   Quote: *[…] The relationship to the patient is also important here, I'm sometimes almost blackmailed, as in: if you don't give me a prescription, then I'll just go see someone else who does. In this case, I try to at least keep the patient with me and continue working with her and see what ... the real reason might be.* (GP male 3 P:131)   - **Individually solutions to keep the treatment with hypnotics or sedatives under control**   *Quote 5: Of course, I always try to make it clear, that frequent use of sleeping pills may diminish their ability to work over time. And I try to convince patients to adopt an interval treatment simply to retain the effectiveness of the medication. (*GP12 male: 24) | - **Treatment with hypnotics and sedatives in regard of sleeping problems is not a matter of major concern**   *Quote 6: Well, in the surgical ward sleeping pills are not significant. It's more of a fringe area for us.* (HD10 male: P8) |
| **Main category: Time resources** | |
| - **Handling is time-consuming. Necessary consultations were not covered.**   *Quote 7: So in the scenario, a patient comes and says I can't sleep, and I simply prescribe a sleeping pill, that's not what I do, I never have (...) But it takes a lot of time that the system does not have.* (GP7 female: P92) | - **Shiftwork determines the handling** - **Ward physicians appreciated the opportunity to act patient-oriented**   Quote: *When I'm on duty in the ward, I see the patients in the morning when I do my round in the ward, then I have some time to get to know him, to see who it is, where does he come from, why's he here, and what are we going to do with him from a medical point of view.* (HD3 male: P67)   - **Physicians on call perceive the handling unsatisfactory**   *Quote 8: (...) sometimes I do it without questioning because of the pressure, the stress and the need to somehow get through the day. Also, things are done against better judgement.* (HD3 male: P39) |
| **Main category: Prescribing options** | |
| - **Out of the SHI** - **Feared recourse claim**   *Quote: Some time ago, we had arranged for this to be generally taken care of through health insurance prescriptions, and we decided that we'd not really like to be subject to their control mechanisms, who knows what ideas the health insurance might come up with. (GP5 male: P55)*   - **Cap their prescribing budget**   *Quote: Of course, that also release my drug budget. (HD12 male P40)*   - **Encourage personal responsibility and self-care**   Quote: *My budgets are limited, that's just the truth of it, so I need to be sure to make prescriptions very carefully, and this just doesn't include sleeping pills*. (GP male 11: P23)   - **The medical indication is missing**   Quote: GP13: *One reason [to prescribe this drug outside the SHI] is the legal requirement, and then the patient is to be reminded quite clearly by this procedure that this is not required from a medical point of view.* (male: P28)   - **Within the SHI**   Quote: *Yes, I prescribe them (in the case of a long-term prescription covered by the health insurance), but for immediate emergencies, I go through health insurance prescriptions as well.* (GP 13 male: P26) | - **For now, as p.r.n medication**   Quote 10. *If it's expected that this truly is a long-term problem that has persisted for some time already, then yes (prescribe continuous medication), but first and foremost, according to the requirement, most of all when I do not know the patient.* (HD6 male: P29)   - **In the case of persistent problems as regular medication**   *Quote 10: If it is likely that it is really a long-term problem, going on over some time [as a chronic prescription], but if I do not know the patient it is primarily based on need* (HD6 male: P27). |
| **Main category: Setting-dependent risk assessments** | |
| - **Limited control of follow up prescriptions**   Quote: *Mountains of repeat prescriptions and that, of course, is a terrible danger when benzo was part of a sleeping pill and is simply continued to be prescribed through this mechanism, and I don't talk with the patient, he doesn't come to see me in my office, and I don't talk to him and ask, how're you doing.* (GP 7 female: 156)   - **No control of increased risk of falling**   *Quote 11: (Physician) I have the impression that we try not to prescribe these medications because they lead to falls. That means that addiction is not really the reason* (GP10 female: 35) | - **p.r.n. medication provoke automatism**   *Quote 12: [In the notes] a prescription medication is written…but no maximum dose is noted but that the patient cannot sleep, and another dose is slipped in, (...) sometimes even without being documented, (...) it's also the case that nurses take it upon themselves to issue these substances.* (HD3 male: P105)   - **In-house-emergencies**   Quote: *umm... most of the incorrect dosages were prescribed in-house in the normal ward, they don't come to us from outside ... Most cases are certainly incorrect dosages ... that is, they are the result of incorrect dosing in the prescription.* (HD5 male: P53)   - **Paradoxical reactions**   Quote: *And I actually had one or two cases where elderly people who took Zolpidem became slightly delirious, dotty, during the night.* (HD10 male P:2)   - **Risk of falls**   Quote: *It's a great problem when they've been newly operated upon and then fall again, and in that case, in these two cases where the healing process was disturbed and another where the result was a periprosthetic fracture, that is always a catastrophe, of course.* (HD8 female: P66)   - **Patients conceal the use**   Quote: *It's a classic, isn't it, people don't tell you because they know, of course, that it's not quite correct what they've been doing, well, and a few days later they are delirious, and if you check the handbag and you're lucky, well, you find it* (HD2 male: P210). |
| **Main category: The role of nurses** | |
| - **Practice nurse as a key link in the prescription chain**   *Quote 13: I think the greatest danger is always that they've been given benzodiazepines twice, and somehow this is then included with the great number of prescriptions passed to them over the counter in the reception area without us General Practitioners having talked with the patients.* (GP7 female: P144) | - **Nurses are the main contact persons**   Quote: *Indeed, and most patients don't ask us doctors, but ask the nurses. The nurses are asked when they are on their last evening round through the ward whether the patient couldn't be issued some sleeping pills*. (HD4 female: P23)   - **Nurses dispense on their initiative**   *Quote 14: Legally, the doctor on duty must have seen the patient to judge whether they are so restless and delirious, that they need a prescription medication (...).In reality, it is unfortunately sometimes the case that a prescription medication has been specified and all is written down from the previous day or nights, and that it is then administered by the nurses without further consultation of the doctor.* (HD7 male: P153)   - **Prescription for agitated patients to reduce the workload of nursing staff**   Quote: *There's the other aspect, of course, that we quite often have very restless and fidgety patients, but then again, it's also our decision here what we tell them ... okay, we've made it easier for the nurses on the way, as it were, and that is also an argument.* (HD6 male: P19) |
| **Main category: The role of colleagues** | |
| - **To handle prescriptions from their predecessor**   Quote: *Regrettably, this is also connected with habits in prescribing medication, which doesn't, ummm, leave us as free in our decisions as we might like because there's habituation effects and the patients find it hard, umm, to let go of a way of life they've become, they've become used to* (GP3 male: 21)   - **To handle prescriptions from their colleagues**   *Quote 15: Of course, there are some cases where we simply continue to prescribe, where we weren’t the ones that made the first prescription (...) there are many extensions to prescriptions from previous doctors.* (GP3 male; P19) | - **Lack of communication between peers**   Quote: *Well, it does occur that I think, gosh, what was he thinking here; there is no need for a prescription of benzos, or something such as that, but this is more a marginal aspect.* (HD3 male: P69) |
