# Supplementary material for: The interplay of context factors in hypnotic and sedative prescription in primary and secondary care—a qualitative study
Source: Eur J Clin Pharmacol. 2018 Sep 13;75(1):87–97. doi: 10.1007/s00228-018-2555-9 (PMC6326988; doi:10.1007/s00228-018-2555-9)
Supplement: Supplementary file 5 — (DOCX 367 kb) [file 228_2018_2555_MOESM5_ESM.docx]

**
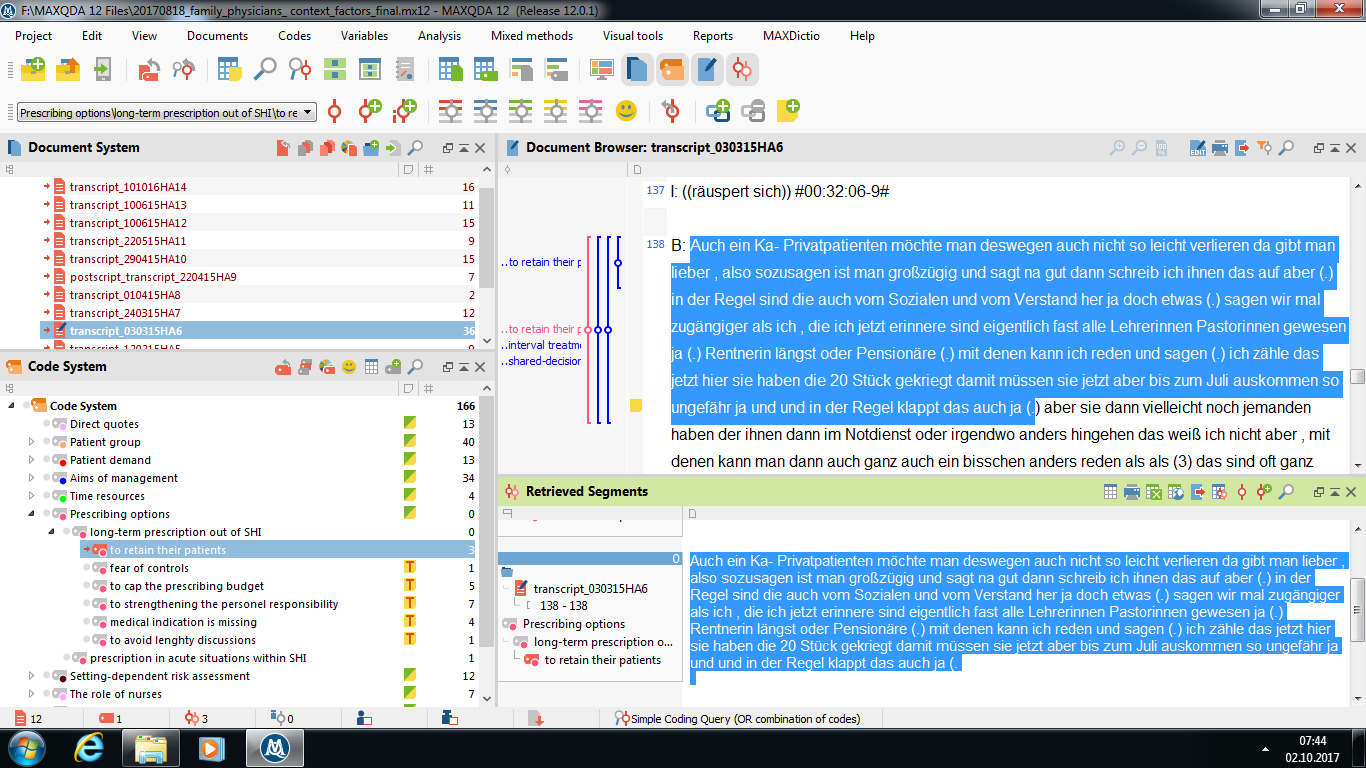
Appendix 5: Excerpt from the data analysis (step 7), using MAXQDA**

Picture 1: Excerpt from the analysis with general practitioners


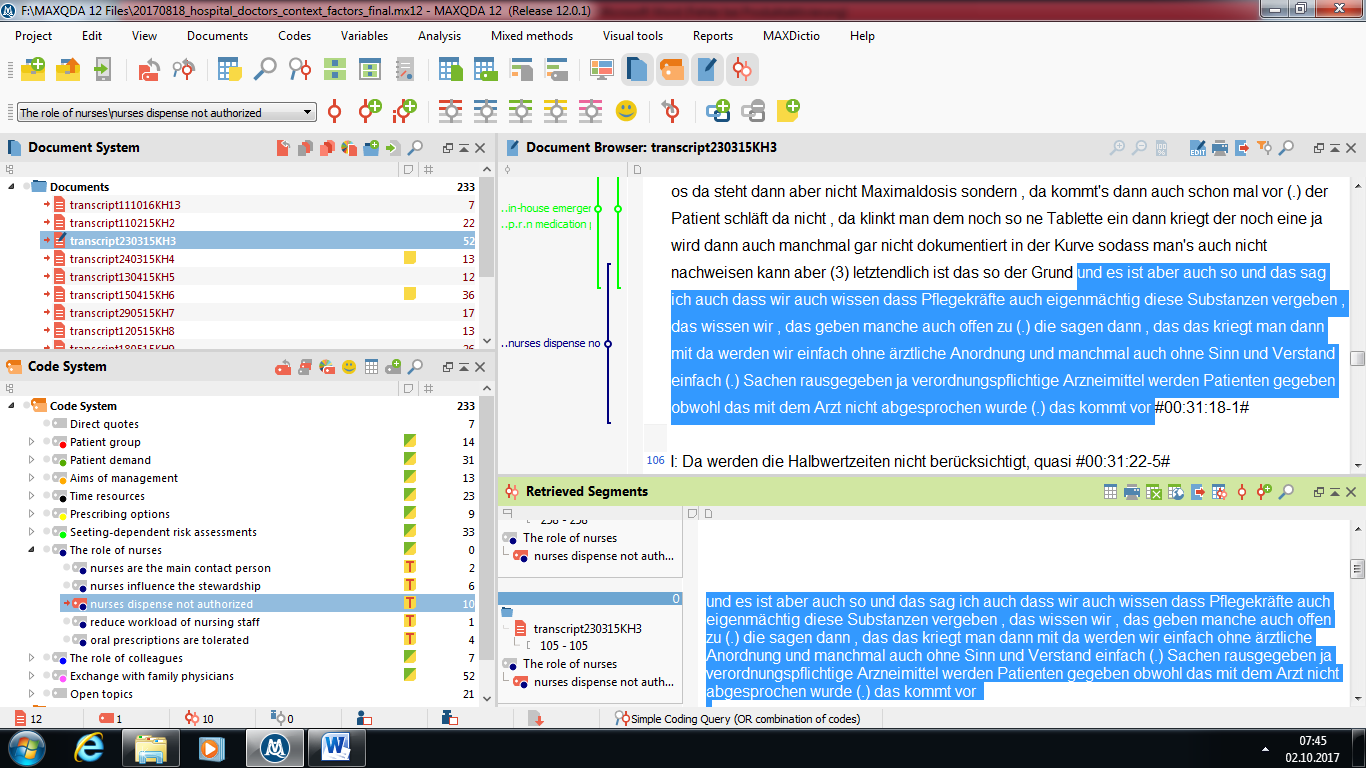


Picture 2: Excerpt from the analysis with hospital doctors
