# Supplementary material for: The interplay of context factors in hypnotic and sedative prescription in primary and secondary care—a qualitative study
Source: Eur J Clin Pharmacol. 2018 Sep 13;75(1):87–97. doi: 10.1007/s00228-018-2555-9 (PMC6326988; doi:10.1007/s00228-018-2555-9)
Supplement: Supplementary file 6 — (DOCX 27 kb) [file 228_2018_2555_MOESM6_ESM.docx]

**Appendix 6: Consolidated criteria for reporting qualitative studies (COREQ): a 32-item checklist for interviews and focus groups.**

Due to the page restrictions, it is not possible to describe all items in detail. For this reason, we fill in the COREQ checklist separately. Please contact us if you need further information.

1. **Which author/s conducted the interview or focus group?**

The first author Vivien Weiß (VW). **(Mentioned in the article)**

1. **What were the researcher’s credentials**

VW is a nurse and studied health management (B.Sc.) and public health (M.Sc.) at the University of Applied Science in Fulda, Germany.

Prof. Dr. Roland Nau, Department of Geriatrcis (Head of the Department), Evangelisches Krankenhaus Göttingen-Weende

Prof. Dr. Gerd Glaeske, Long-Term Care and Pension (Head of the Department Health), University of Bremen, SOCIUM Research Center on Inequality and Social Policy

Prof. Dr. med. Eva Hummers, Head of the Department of General Practice at the University Medical Center Göttingen, Germany.

Prof. Dr. Wolfgang Himmel, Sociologist, Department of General Practice at the University Medical Center Göttingen, Germany

1. **What was their occupation at the time of the study?**

VW was and is a PhD-Candidate and a research assistant at the Department of General Practice at the University Medical Center Göttingen, Germany.

1. **Was the researcher male or female?**

Female

1. **What experience or training did the researcher have?**

VW studied public health and learned to carry out qualitative studies. Explicit for this study she visited two workshops for qualitative content analysis in Magdeburg, February 27, 2015, and in Göttingen, September 16, 2016.

1. **Was a relationship established before study commencement?**

VW had no relationship with any of the participants. VW invited all general practitioners via letter or telephone and all hospitals doctors of the regional hospital via email to take part in the study. To recruit the hospital doctors, we also used the internal news service of the hospital. The second contact was established by phone. The interviews were conducted face-to-face.

1. **What did the participants know about the researcher?**

During the telephone call before the interview, VW had introduced herself as nurse and research assistant working at the Department of General Practice at the University Center. Participants reached an information sheet and consent form.

1. **What characteristics were reported about the interviewer/facilitator?**

It was reported that the authors might have pre-existing perceptions due to their occupation and experiences. Therefore the data was also discussed with non-healthcare providers. **(Mentioned in the article: data analysis)**

1. **What methodological orientation was stated to underpin the study?**

We used qualitative methods to generate broad descriptions of the prescribing processes, motives, and experiences in primary and secondary care and to understand better the influence of contextual factors on the ‘game’ of prescribing hypnotic and sedative drugs. **(Mentioned in the article)**

1. **How were participants selected?**

All hospital doctors from the participating hospital were invited to take part. An attempt was made to interview at least one person from all departments and professional positions. Furthermore, a subsample of 46 general practitioners in Lower Saxony and Northern Hesse were invited to take part. We tried to stratify the participants by age, gender, and urban vs. rural setting. The participants were selected according to maximum variation. **(Mentioned in the article)**

1. **How were participants approached?**

A few days after the written invitations, a phone call by VW was made to establish contact. In one case a study nurse contacted a general practitioner to take part in the study.

1. **How many participants were in the study**?

In total, the sample consisted of 12 (3 females) hospital doctors and 12 (5 females) general practitioners. The hospital doctors comprised different departments and hierarchical positions: geriatrics (n=3), emergency medicine (n=2), internal medicine (n=3), general surgery (n=2) and trauma surgery (n=2); chief physicians (n=1), senior physicians (n=6) and assistant physicians (n=5). The sample of general practitioner worked in urban areas (n=7, two females) and rural areas (n=5, three females).

1. **How many people refused to participate or dropped out? Reasons?**

We invited all hospital doctors in the participating regional general hospital via email and within the internal news service. After an additional invitation of a subsample of 21 hospital doctors according to the hierarchical position, gender and department, a total of 10 doctors agreed to participate. We also invited per letter, fax and telephone contact a sample of 46 general practitioners stratified by age, gender, and urban vs. rural setting from Lower Saxony and Northern Hesse, Germany; 10 of them agreed to participate. (2) After a preliminary analysis of the first interviews in both settings, we deliberately addressed some more doctors with characteristics that seemed to be important to understand the scope of experiences fully and to obtain maximum variance within the sample. Thus, two more female hospital doctors from internal medicine and general surgery of the participating hospital and two younger general practitioners were invited. Reasons for non-participation were no interest or no time, but this was not systematically assessed. **(Mentioned in the article)**

1. **Where was the data collected? e.g., home, clinic, workplace**

According to participants´ choice: the interviews with general practitioners were all conducted in the practitioners’ practices; the interviews with hospital doctors were conducted in the hospital in extra rooms, in offices or the cafeteria.

1. **Was anyone else present besides the participants and researchers**?

No.

1. **What are the important characteristics of the sample?**

Time of the data collection, gender of the participants, the professional positions and the departments or number of urban/rural practices are **mentioned in the article**. Data reporting years of experience and participants ‘qualification was not reported here due to length restrictions.

1. **Were questions, prompts, guides provided by the authors? Was it pilot tested?**

The interview guideline was developed and tested in 4 pilot interviews with general practitioners and hospital doctors who were not included in the sampling frame. The topics of the guideline were drawn from current literature and a previous quantitative survey and were informed by the experience of clinical members of the research team. **(Mentioned in the article)**.

1. **Were repeat interviews carried out? If yes, how many?**

No.

1. **Did the research use the audio or visual recording to collect the data?**

All the interviews were conducted by VW and occurred between January 2015 and October 2016. They lasted half an hour on average (range: 17 to 56 minutes) and were audio-recorded and transcribed verbatim. **(Mentioned in the article).**

1. **Were field notes made during and after the interview or focus group?**

VW made written notes during the interview and wrote postscripts and case summaries for the later data analysis. Furthermore, VW wrote memos during the data analysis process.

1. **What was the duration of the interviews or focus group?**

They lasted half an hour on average (range: 17 to 56 minutes) (**Mentioned in the article).**

1. **Was data saturation discussed?**

The data saturation was reached at the time when no new information could be collected **(Mentioned in the article).**

1. **Were transcripts returned to participants for comment and correction?**

No.

1. **How many data coders coded the data?**

Primarily VW; the codes were discussed with Lea Heuckeroth, in two workshops with researchers who were not involved in the project and with the research team with expertise in hospital geriatrics, family medicine, nursing science, health services research and medical sociology **(mentioned in the article).** Preliminary results were presented orally at the 9th European Public Health Conference Vienna, Austria, November 11, 2016.

1. **Were themes identified in advance or derived from the data?**

We analyzed all the interviews by the summarizing qualitative content analysis and coded the material by combining data-driven and concept-driven strategies. That means, primarily the themes identified were derived from the data. In a sixth step, the categories were ordered following the definition of ‘external context factors,’ as defined by Helman [22], including the physical place or setting, equipment, atmosphere, type of relationship, status, and type of information. In a final step, the categories were summarized at a higher level as ‘context factors,’ using Bourdieu's concept of ‘theory of practice.’ **(Mentioned in the article and see Appendix 3).**

1. **What software, if applicable, was used to manage the data?**

MAXQDA 12 **(mentioned in the article and see Appendix 5).**

1. **Did participants provide feedback on the findings?**

Not yet.

1. **Were participant quotations presented to illustrate the themes/findings?**

Yes, see table 1 and appendix 4.

1. **Was each quotation identified? e.g., participant number**

Yes, in the article and in appendix 4.

1. **Was there consistency between the data presented and the findings?**

The validity and usefulness of themes and subthemes were regularly evaluated by the research team to ensure consistency and coherency. **(Mentioned in the article)**

1. **Were major themes presented in the findings?**

Yes, see **the results in the article.**

1. **Is there a description of diverse cases or discussion of minor themes?**

See **Discussion: comparison with literature:**

- The significance of risk of falls for hospital doctors and general practitioners
- Influence in prescribing of other colleagues
